# Genome-wide transcriptional profiling data from chronic cutaneous lupus erythematosus (CCLE) peripheral blood

**DOI:** 10.1016/j.dib.2014.11.006

**Published:** 2014-12-11

**Authors:** R. Dey-Rao, A.A. Sinha

**Affiliations:** Department of Dermatology, University at Buffalo, 6078 Clinical and Translational Research Center, 875 Ellicott Street, Buffalo, NY 14203, United States

**Keywords:** LE, CCLE Chronic cutaneous lupus erythematosus, NL, Normal control, B, Blood

## Abstract

The disease Lupus erythematosus (LE), exhibits a variety of clinical manifestations with potentially wide-ranging multi-organ damage to joints, tendons, kidney, lung, heart, blood vessels, central nervous system and skin [1,2] Systemic changes are likely to trigger organ specific manifestation of the disease. Here, we provide the data examined to address the gap in knowledge regarding causes and mechanisms contributing to the autoimmune attack on skin in chronic cutaneous lupus erythematosus (CCLE). The raw gene expression data files (CEL files) are provided with this article [3].

## Specifications table

**Table:** 

Subject area	Dermatology
Organism/cell	Homo sapiens
More specific subject area	Chronic cutaneous lupus erythematosus (CCLE) blood samples
How data was acquired	Affymetrix GeneChip HG-U95set
Data format	Raw data: CEL files
Experimental factors	CCLE patients and healthy control comparison
Experimental features	We performed both unsupervised and supervised analysis and established differentially expressed genes (DEGs) between CCLE patient and healthy control blood samples. The DEGs were then subjected to a pathway and biological processes-based enrichment analyses in DAVID and Metacore
Data source location	Patients diagnosed with CCLE and more specifically, DLE were recruited into the study from the Dermatology Outpatient Clinic of New York Presbyterian Hospital, Cornell University
Data accessibility	Data is available with this article

## Value of the data

• The raw genome-wide transcriptional profiling data from CCLE and healthy patients are provided here as CEL files and are available for further analysis.
• The data fill a major gap in knowledge regarding the systemic changes that underlie skin specific manifestations in cutaneous lupus.
• The data can be used to link differential gene expression to a broad range of biological processes and pathways underlying the mechanism of CCLE.

## 1 Data, materials and methods

The raw data files (CEL files) that were used in the analysis and interpretation in [3] are available in Supplementary information.

Sample IDs are:

LE1008B
LE1009B
LE1011B
NL1004B
NL1013B
NL1014B.

We used the Affymetrix Human Genome U95set GeneChip according to standard Affymetrix protocols to analyze gene expression from blood samples of CCLE patients (*n*=3) and healthy control individuals (*n*=3). We used Affymetrix expression console to check the quality of each individual CEL file. Gene expression data was imported into Partek Genomic Suite v 6.6 as CEL files using default parameters. Preprocessing: RMA, including log 2 transformation, quantile normalization, background correction and median polish probe set summarization to bring mean expression values of all 6 arrays to the same scale. We performed both unsupervised and supervised hierarchical cluster analysis and established differentially expressed genes (DEGs). Pathway and biological processes-based analyses were performed via enrichment analyses in DAVID and Metacore. Details of methodology used are provided in materials and methods and supplementary materials and methods [3].

## Footnotes

Appendix A Supplementary data associated with this article can be found in the online version at doi:10.1016/j.dib.2014.11.006.
